# Study protocol: the Labor Progression Study, LAPS - does the use of a dynamic progression guideline in labor reduce the rate of intrapartum cesarean sections in nulliparous women? A multicenter, cluster randomized trial in Norway

**DOI:** 10.1186/s12884-017-1553-8

**Published:** 2017-11-13

**Authors:** Stine Bernitz, Rebecka Dalbye, Pål Øian, Jun Zhang, Torbjørn Moe Eggebø, Ellen Blix

**Affiliations:** 1 Department of Obstetrics and Gynecology, Østfold Hospital Trust, PO.box 300, 1714 Grålum, Norway; 2 Department of Health, Nutrition and Management, Faculty of Health Sciences, Oslo and Akershus University College of Applied Sciences, Oslo, Norway; 3 Department of Obstetrics and Gynecology, The University Hospital of north Norway, Tromsø, Norway; 4 Institute of Clinical Medicine, Faculty of Health Sciences, University of Tromsø, Tromsø, Norway; 5 Xinhua Hospital, Shanghai Jiaotong University School of Medicine Shanghai, China, National Center for Fetal Medicine, Shanghai, China; 6 Trondheim University Hospital (St Olavs Hospital), Trondheim, Norway; 7 Department of Laboratory Medicine, Children’s and Women’s Health, Norwegian University of Science and Technology, Trondheim, Norway

## Abstract

**Background:**

The increasing rate of intrapartum cesarean sections is subject of attention and concern as it is associated with adverse outcomes. Labor dystocia is one of the most frequent indications for cesarean sections even though there is no consensus on criteria for labor dystocia. Traditionally the progression of labor follows guidelines based on Friedman’s curve from the mid 1950s. In 2010 Zhang presented a new labor curve and a dynamic guideline for labor progression based on contemporary research. The main aim of this trial is to evaluate whether adhering to Zhang’s guideline for labor progression, changes the intrapartum cesarean section rate in nulliparous women without jeopardising maternal and neonatal outcomes compared to a traditional guide line called the 4-h action line based on Friedman’s curve.

**Methods/design:**

A multicenter cluster randomized trial including 14 birth care units in Norway is conducted. Seven units are randomized to use the 4-h action line guideline for labor progression and seven units are randomized to use Zhang’s new dynamic guideline for labor progression, for all nulliparous women with a singleton fetus in a cephalic presentation and spontaneous onset of labor at term. Clinical outcomes are compared between the groups. The determination of the sample size (number of clusters and individuals) is based on a power calculation of intrapartum cesarean section, which is 9.2% in the study population (p1). Further, we expect that the intrapartum cesarean section rate will be 6.7% (p2) which is a 25% reduction, when using the new guideline. With a chosen significance level of 0.05, a power of 80% and p1 = 9.2% and p2 = 6.9%, we should include at least 14 clusters and 6582 individuals.

**Discussion:**

Clinical consequences when using the guideline by Zhang have, to the best of our knowledge, not been investigated earlier. The results will provide a strong basis to make a qualified decision on whether it is beneficial to introduce a dynamic labor progression curve in contemporary obstetrics both nationally and internationally.

**Trial registration:**

Clinicaltrials, NCT02221427

## Background

The increasing rate of intrapartum cesarean sections in high resource countries is subject of attention and concern as it is associated with adverse outcomes for mother and infant [1]. Intrapartum cesarean sections that neither benefit mother nor infant should be avoided. An uncomplicated vaginal birth in nulliparous women strongly predicts uncomplicated labors and deliveries in subsequent pregnancies [2], while intrapartum cesarean sections are associated with a 50% subsequent cesarean section rate [3] and adverse outcome for the mother [4–6] and for the baby [7, 8]. The economic aspect of labor is also of importance as an intrapartum cesarean section is significantly more expensive compared to a spontaneous vaginal delivery [9]. The rate of cesarean sections for nulliparous women with a singleton fetus in a cephalic presentation and spontaneous onset of labor at term has increased from 5.7% to 9.2% in Norwegian hospitals (2000–2012) [10]. The most common indication for intrapartum cesarean sections is labor dystocia [11] even if there is no consensus on criteria for the diagnosis.

Labor dystocia is characterized by abnormal slow progress of labor and is among the most common challenges of birth care especially in nulliparous women [12, 13]. When labor progression is assessed to be prolonged according to current guidelines, labor dystocia is primarily treated by amniotomy followed by oxytocin infusion to augment the contractions of the uterus. Oxytocin is a potent drug and is classified by the Institute for Safe Medication Practices in the USA as one of 12 medications which is “bearing heightened risk of harm” [14]. Even if used correctly, oxytocin is associated with increased risk of instrumental vaginal delivery, episiotomy [15], intrapartum cesarean section, low Apgar score, low pH in neonates and increased risk of transfer to a neonatal intensive care unit [16]. If augmentation with oxytocin does not lead to adequate effect, a cesarean section is often performed.

Nulliparous women with a singleton fetus in a cephalic presentation and spontaneous onset of labor at term are often considered to be low-risk and have considerable potential of spontaneous labor without interventions. Despite this fact, the rate of oxytocin augmentation in low-risk nulliparous women is unreasonably high. Over the last decade the rate of oxytocin augmentation has been reported to be from 44 to 75% in eg Norway, Sweden and Brazil [15, 17–20]. It has not been proved that the high rates of oxytocin reduce the cesarean section rate or improve birth outcome for mother or baby [12, 21].

The labor process is divided in two stages; the first stage and the second stage. The first stage is divided in two phases; the latent phase and the active phase. The latent phase starts when the woman experience regular and painful contractions until the cervix is dilated four centimetres. The active phase is defined from a cervix dilatation of four centimetres until full dilatation. The second stage is divided in two phases; the descending phase where the baby’s head is descending towards the pelvic floor and the expulsion phase where the mother is actively pushing the baby out [21, 22].

**Fig. 1:**
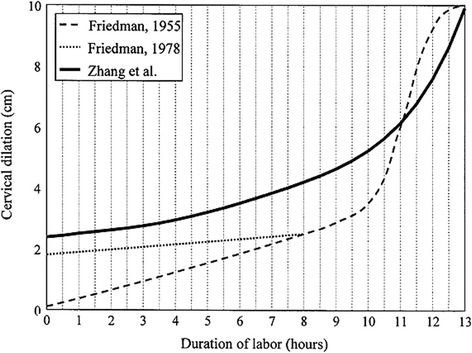
Labor progression curve in first time mothers presented in cervical dilatation. Zhang [28]. Reprinted with permission from Elsevier and Copyright Clearance Center (4104651193)

In Norway the guidelines for expected progression of labor is based on interpretations of Friedman’s curve of cervical dilatation (Fig. 1), a linear labor progression curve developed in 1953 [23]. Contemporary research by Zhang shows that the dilatation of the cervix can be substantially slower than earlier expected, especially at an early stage of labor [24, 25] (Table 1).

**Table 1:** Expected time intervals from cm to cm presented in hours and minutes (50 percentile and 95 percentile) according to Zhang et al. [24]

Dilation of the cervix	50 percentile^a^	95 percentile^a^ (dystocia)
From 4 to 5 cm	1 h / 15 min	6 h / 30 min
From 5 to 6 cm	45 min	3 h / 15 min
From 6 to 7 cm	30 min	2 h / 15 min
From 7 to 8 cm	30 min	1 h / 30 min
From 8 to 9 cm	30 min	1 h / 30 min
From 9 to 10 cm	30 min	1 h / 45 min
2. stage without EDA^a^ Expulsion phase max 60 min.	30 min	2 h / 45 min
2. stage with EDA Expulsion phase max 60 min.	1 h	3 h / 30 min
Total time without EDA	4 h / 30 min	19 h / 30 min
Total time with EDA	5 h	20 h / 15 min

^a^EDA (epidural analgesia)

### Labor progression curves

To visualize the progression of labor, the status of the cervix dilatation is recorded throughout labor on a graphic curve called a partogram. The partogram is widely used in Norway and also internationally and facilitates that midwives’ and doctors’ can monitor labor progression, and together with criteria for labor dystocia, consequently carry out necessary interventions.

**Fig. 2:**
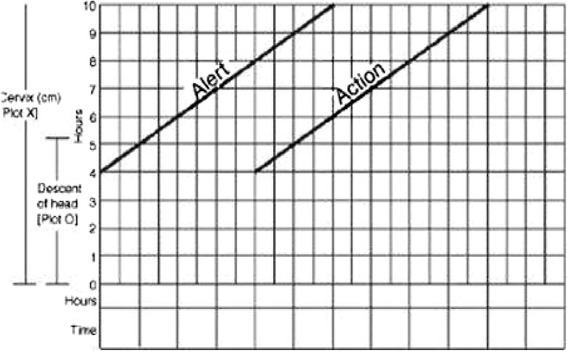
Illustration of the 4 h action line with an alert line (expected progression) and an action line (labor dystocia if crossed) according to Philpott et al. [27]

In the nineteen fifties Friedman presented the first progression curve based on examination of 100 nulliparous women (Fig. 1) [23]. In the late seventies, Friedman presented the curve starting from 2 cm of cervical dilatation [26]. Friedman’s curve has been widely adopted and applied internationally for more than 60 years. The curve is the basis for what is called a “four-hour action line” (Guideline F, Fig. 2) which is a guideline to diagnose labor dystocia presented by Philpott in 1972 [27]. In 2002 Zhang presented a new labor curve based on data from 1329 low-risk women [28]. Zhang’s findings were confirmed in a large cohort of 26,838 women in 2010 (Fig. 1) [24].

A new guideline (Guideline Z, Table 1) for normal birth progression is developed according to Zhang’s curve and contemporary research findings [24]. The new guideline differs markedly from the Friedman’s curve in that the cervix dilates substantially slower, especially before reaching six centimetres of dilatation, nor is the distinct deflection of the curve between nine and 10 cm in Friedman’s curve found in Zhang’s curve (Fig. 1). These findings may suggest that the diagnostic criteria for labor dystocia are too stringent following Friedman’s curve (Fig. 1). The new guideline also differs from the existing ones by not expecting a linear progression and by defining the progression to be normal if the speed of cervical dilatation is within the 95 percentile.

There is no worldwide consensus on duration of spontaneous labor and therefore no consensus on how to define labor dystocia. The duration of labor and labor curve patterns are still subjects of discussion [29]. Clinical consequences when using the guidelines and progression curve by Zhang have, to our knowledge, not been investigated with rigorous study design. In this trial we want to compare the cesarean section rate in nulliparous women based on whether they adhere to a guideline based on Friedman’s progression curve or to Zhang’s guideline for labor progression, in active labor. In each case, there will be recorded whether labor dystocia was diagnosed according to the allocated guideline.

### Hypothesis

By adhering to the guideline for labor progression presented by Zhang [24], the rate of intrapartum cesarean sections in nulliparous women will decrease compared to adhering to the guideline for labor progression based on Friedman’s curve [23], without jeopardizing maternal and neonatal outcomes.

## Methods and design

To test the hypothesis, a cluster randomized trial is conducted. Fourteen clusters (birth care units) from all four health regions in Norway are included. Birth care unit inclusion criteria: Units willing to adhere to the guidelines in the trial period and who consider that they have the capacity to participate both logistically and practically. Birth care units with more than 500 deliveries per year to secure a reasonable inclusion period.

Guideline F = The four hour action line: Guideline with the following expected labor progression: if the cervix dilates at least 1 centimetre (alert line) per hour assessed after 4 hours (action line). Labor dystocia is diagnosed if progression proceeds slower than this definition throughout the active phase of the first stage of labor, i.e. if the action line is crossed. Labor dystocia in the second stage of labor is diagnosed if the descending phase lasts longer than one hour, two hours for women with epidurals or if the expulsion phase lasts longer than 60 minutes. This guideline is based on an interpretation of Friedman’s expected progression of labor (Figs. 1 & 2).

Guideline Z = Zhang’s guideline: Dynamic guideline which takes into account the dilatation of the cervix on admission and calculates the expected progression during the active phase of the first stage of labor according to time intervals from cm to cm. Labor dystocia is diagnosed if the time intervals according to the 95 % percentile are exceeded. Labor dystocia in the second stage of labor is diagnosed if the descending phase lasts longer than one hour and 45 minutes, two hours and 30 minutes for women with epidurals or if the expulsion phase lasts longer than 60 minutes. This guideline is based on the labor progression curve by Zhang (Fig. 1 & Table 1).

If labor dystocia occurs, either by crossing the action line in arm F, or if the time intervals are exceeded in arm Z, national guidelines for labor arrest will be followed: If the membranes are not ruptured, an amniotomy will be performed, and if there is still no progression after 1 h, oxytocin will be given. If the water is already broken, oxytocin will be given at the point of labor dystocia. The national guideline according treatment of labor dystocia suggest a starting dose of 30 ml/h (=300 mU/h) of 10 ie oxytocin mixed in 1000 ml intravenous fluid and a raise of 15 ml/h (150 mU/h) until satisfactory number of contractions, or until the maximumdose of 180 ml/h (1800 mU/h) is reached [30]. Individual assessments will of course be made to secure safe birth care.

Half of the clusters are randomized to adhere to either guideline F or Z (presented above) in active labor for all nulliparous women with a singleton fetus in a cephalic presentation and spontaneous onset of labor at term. These women are often denoted as group I, according to the “ten group classification system” (TGCS) published by Robson [31]. Active labor is defined when the cervical dilatation is 4 cm or more until the baby is born.

The choice of cluster randomizing, which means randomizing at a hospital level, is due to the risk of contamination if randomized on an individual level. Key elements to specify regarding allocation of treatment are: The method of generating the allocation sequence was computer-generated, the allocation ratio was equal to one, and the type of randomization was restricted and the factors “size of birth care unit” and “prior rates of cesarean sections for nulliparous women with a singleton fetus in a cephalic presentation, in spontaneous onset of labor at term” was used for stratification.

The randomization process was performed through a central computer assisted program. The trial will be conducted according to the CONSORT statement for planning and implementation of cluster randomized trials [32].

All clusters have read and approved the trial protocol and signed the cooperation agreement. Each cluster provides one dedicated person (local coordinator) responsible for the trial during the inclusion period. The local coordinator is responsible for recruitment and inclusion of participants, and monitoring the entries in a web-Case Report Form (web-CRF). Local coordinators will cooperate with the principle investigator throughout the study period and must report any challenges to the principal investigator.

### Enrolment of participants

All women in TGCS group I will adhere to the labor progression guideline that the cluster (birth care unit) is randomized to. All nulliparous women planning to give birth at any of the included birth care units receive written information about the trial when called for the second trimester ultrasound examination or at the labor ward. Eligible women are asked to sign an informed consent permitting her data to be included in the analyses, and to answer an on-line questionnaire about her childbirth experience (Childbirth Experience Questionnaire, CEQ) [33].

#### Safety assessment

All women in both arms will be cared for and monitored according to medical guidelines at each birth care unit. Necessary interventions due to the women’s or the fetus’ needs, will be conducted regardless of the allocated guideline for labor progression.

### Primary endpoint

The rate of intrapartum cesarean sections.

### Secondary endpoints

Indications for intrapartum cesarean sections, the rate of oxytocin augmentation during labor including indication for initiating oxytocin infusion, cervical dilatation at onset of augmentation with oxytocin and the total duration of augmentation with oxytocin. Time related aspects of labor progress including latent phase, active phase of first stage, latent phase of second stage, expulsion phase and second stage in total. The use of epidural analgesia. The rate and mode of fetal monitoring during labor.The rate of: dystocia, instrumental vaginal deliveries, episiotomies, anal sphincter injuries, women with post partum hemorrhage (< 500 ml, 500–1000 ml, and > 1000 ml), blood transfusions, children with Apgar score ≤ 7 after 5 min, children with umbilical cord pH ≤ 7.05 and ≤ 7.0 and Base Deficit > 12 mmol/l, transfers of neonates to the Neonatal Intensive Care Unit including indication for transfer. Women’s experience with birth care will be investigated using “The Childbirth Experience Questionnaire, CEQ” [33] which will be sent to each participant approximately 4 weeks after discharge.

### Web-case report form

Due to different systems for electronic medical records and due to additional handwritten records, a web based Case Report Form (web-CRF) has been designed by the Unit of Applied Clinical Research at the Faculty of Medicine, the Norwegian University of Science and Technology, NTNU [34] customized for this study. The web-CRF is transferable to the analytical tools; STATA and SPSS. Local coordinators at all clusters have access to the web-CRF which contain boxes for all required information. The system is 100% traceable which allows access to all corrections with dates and signatures.

### The Childbirth Experience Questionnaire (CEQ)

To investigate possible differences in birth experience between the two groups, women’s experiences of birth care will be surveyed. The CEQ is a multidimensional validated instrument to evaluate women’s experience with birth care [33] and is distributed to participating women.

The LAPS research group has obtained permission from the developers of the CEQ to use it in this study. All women who have entered their e-mail address on the consent form will receive an e-mail with an online login ID to answer the web-based questionnaire. The online version of the questionnaire is developed at the Department for Research Computing (USIT), the University of Oslo, Norway [35] and the data are stored at the Services for Sensitive Research Data at the same unit.

### Implementation

The local coordinators appointed at each participating birth care unit will strive to ensure that the units adhere to the allocated guideline. The local coordinators are also responsible for assuring that data entered into the web-CRF are complete, accurate, and that entry is performed in a timely manner. The signature of the local coordinators will attest to the accuracy of the data on each web-CRF.

Data will be recorded both in the electronic medical record and on paper when it comes to the partograms by the midwife responsible for the birth. Consecutive assessments recorded electronically throughout labor and assessments recorded on the printed version of the partogram will be transmitted to the web-CRF by the local coordinators at each birth care unit. Assessments to be recorded throughout labor are: Cervix status on admission (in centimetres), results of regularly vaginal exploration, use of oxytocin, pain relief and additional interventions.

The records of each participant will clearly describe:

- That the woman is participating in the trial by including the enrolment number and the cluster code
- A statement that she signed the Informed Consent form
- Results of assessments confirming her eligibility for the trial

The principal investigator will be available to the local coordinators and birth care units throughout the trial period to follow up and assist the local coordinators when needed.

The principal investigator will administer and monitor the childbirth experience questionnaire, by ensuring that all registered e-mail addresses receive a CEQ-survey, a reminder and keeping control of incoming surveys.

### Statistical methods and data analysis

The determination of the sample size (number of clusters and individuals) is based on a power calculation with the least occurring outcome; intrapartum cesarean section, which is 9.2% in the study population (p1). Further, we expect that the intrapartum cesarean section rate will be 6.7% (p2) which is a 25% reduction, when using the new guideline. Formula (4) on page 320 in the article by Hayes et al. [36] is used to calculate the needed number of clusters and participants. According to this formula, with a chosen significance level of 0.05, a power of 80% and p1 = 9.2% and p2 = 6.9%, we should include at least 14 clusters and 6582 individuals. The design allows flexibility, so that larger birth care units may contribute with more study subjects than smaller units.

The difference between the randomized groups will be presented with a Risk Ratio (RR) and a 95% confidence interval (95% CI). For dichotomous efficacy variables a significance test taking into account the cluster structure of the data, will be used [37]. For continuous data independent sample t-tests and parametric tests will be used when appropriate. Statistical analysis will be conducted using STATA version 10.1 StataCorp, Texas, //845 USA and SPSS version 18. The analyses will be conducted according to the principle of intention-to-treat.

### Publication policy

When the quality control of the data is conducted, the production of articles will commence. All personnel who have contributed significantly with the planning and performance of the study according to the Vancouver convention 1988 may be included in the list of authors.

### Ethical and regulatory requirements

The trial is and will be conducted in accordance with ethical principles that have their origin in the Declaration of Helsinki and are consistent with Good Clinical Practice and applicable regulatory requirements. Registration of participant’s data will be carried out in accordance with national personal data law. The protocol, including the patient information and informed consent form to be used, is approved by the Regional Committee for Medical and Health Research Ethics: 2013/1862/REK South-East. The principal investigator is responsible for informing the ethics committee of any serious and unexpected adverse events and/or major amendments to the protocol as per national requirements.

The protocol has been approved and signed by the management at the birth care units before commencement of the trial. The protocol was registered in www.clinicaltrials.gov before enrolment of participants NCT02221427.

## Discussion

Even if there is no consensus on how to define labor dystocia, slow progress or arrest is one of the most common indication for intrapartum cesarean sections [11]. Assessment of labor progression is therefore of interest to improve labor care [38]. In this multicenter cluster randomized trial we aim to evaluate if adhering to a dynamic labor progression curve will change the rate of intrapartum cesarean sections compared to adhering to a linear progression curve in active labor for nulliparous women. Nulliparous women with a singleton fetus in a cephalic presentation and spontaneous onset of labor at term, TGCS group I, are included. TGCS group I represents the majority of all nulliparous women (approximately 70%) including all categories regarding age, height weight or conditions. This wide inclusion of participants represents a pragmatic approach to the design which allows a strong generalizability to the population in general.

This present study is already well established at all 14 included clusters. It’s a challenge to secure that all clusters adhere to the allocated guideline, hence; it is of great importance to strengthen the process by continuously follow up the implementation at all sites with guidance and teaching. Drop outs will be recorded to document if eligible women are not included in the study and reason for drop out.

The foundation of knowledge derived from this study may provide a strong basis to make a qualified decision on whether it is beneficial to introduce a dynamic labor progression curve in obstetrics both nationally and internationally. Knowledge about labor progression as a dynamic process will lead to correct diagnosis and better management of an actual prolonged labor, which in turn may lead to a decrease in unnecessary interventions, which will benefit the population both medically and economically [9, 39]. The results from this study may give the basis for developing a new dynamic guideline for labor progression in the electronic obstetric record systems.

## Abbreviations

CEQ: The Childbirth Experience Questionnaire
Guide line F: The 4 h action line with an expected cervical dilatation of 1 cm/h assessed after 4 h. Labor dystocia is diagnosed if the action line is crossed.
Guide line Z: Zhang’s guide line for labor progression with an expected cervical dilatation according to given time intervals from cm to cm. Labor dystocia is diagnosed if the given time intervals are exceeded.
TGCS: The Ten Group Classification System classifies women into 10 groups based on their obstetric characteristics. The groups are totally inclusive and mutually exclusive.
web-CRF: Web based Case Report Form

## References

[1] Bragg F, Cromwell DA, Edozien LC, Gurol-Urganci I, Mahmood TA, Templeton A, van der Meulen JH (2010). Variation in rates of caesarean section among English NHS trusts after accounting for maternal and clinical risk: cross sectional study. BMJ (Clinical research ed).

[2] Mawdsley SD, Baskett TF (2000). Outcome of the next labour in women who had a vaginal delivery in their first pregnancy. BJOG.

[3] Kolas T, Hofoss D, Daltveit AK, Nilsen ST, Henriksen T, Hager R, Ingemarsson I, Oian P (2003). Indications for cesarean deliveries in Norway. Am J Obstet Gynecol.

[4] Jackson S, Fleege L, Fridman M, Gregory K, Zelop C, Olsen J (2012). Morbidity following primary cesarean delivery in the Danish National Birth Cohort. Am J Obstet Gynecol.

[5] Lobel M, DeLuca RS (2007). Psychosocial sequelae of cesarean delivery: review and analysis of their causes and implications. Soc Sci Med (1982).

[6] Smith GC, Pell JP, Dobbie R (2003). Caesarean section and risk of unexplained stillbirth in subsequent pregnancy. Lancet.

[7] Levine EM, Ghai V, Barton JJ, Strom CM (2001). Mode of delivery and risk of respiratory diseases in newborns. Obstet Gynecol.

[8] Renz-Polster H, David MR, Buist AS, Vollmer WM, O'Connor EA, Frazier EA, Wall MA (2005). Caesarean section delivery and the risk of allergic disorders in childhood. Clin Exp Allergy.

[9] Bernitz S, Aas E, Oian P (2012). Economic evaluation of birth care in low-risk women. A comparison between a midwife-led birth unit and a standard obstetric unit within the same hospital in Norway. A randomised controlled trial. Midwifery.

[10] Medical Birth Registry of Norway. Norwegian Institute of Public Health; 2012. http://www.statistikkbank.fhi.no/mfr/. Accessed 1 Nov 2012.

[11] Neal JL, Lowe NK, Patrick TE, Cabbage LA, Corwin EJ (2010). What is the slowest-yet-normal cervical dilation rate among nulliparous women with spontaneous labor onset?. J Obstet Gynecol Neonatal Nurs.

[12] Bugg GJ, Siddiqui F, Thornton JG (2011). Oxytocin versus no treatment or delayed treatment for slow progress in the first stage of spontaneous labour. Cochrane Database Syst Rev (Online).

[13] Kjaergaard H, Olsen J, Ottesen B, Nyberg P, Dykes AK (2008). Obstetric risk indicators for labour dystocia in nulliparous women: a multi-centre cohort study. BMC Pregnancy Childbirth.

[14] Clark SL, Simpson KR, Knox GE, Garite TJ (2009). Oxytocin: new perspectives on an old drug. Am J Obstet Gynecol.

[15] Bernitz S, Oian P, Rolland R, Sandvik L, Blix E. Oxytocin and dystocia as risk factors for adverse birth outcomes: a cohort of low-risk nulliparous women. Midwifery. 2014;30(3):364–70. 10.1016/j.midw.2013.03.010 23684697

[16] Oscarsson ME, Amer-Wahlin I, Rydhstroem H, Kallen K (2006). Outcome in obstetric care related to oxytocin use. A population-based study. Acta Obstet Gynecol Scand.

[17] Blix E, Pettersen SH, Eriksen H, Royset B, Pedersen EH, Oian P (2002). Use of oxytocin augmentation after spontaneous onset of labor. Tidsskr Nor Laegeforen.

[18] Carmo Leal M, Pereira AP, Domingues RM, Theme Filha MM, Dias MA, Nakamura-Pereira M, Bastos MH, Gama SG (2014). Obstetric interventions during labor and childbirth in Brazilian low-risk women. Cad Saude Publica.

[19] Moen MS, Holmen M, Tollefsrud S, Rolland R (2005). Low-risk pregnant women in an obstetric department--how do they give birth?. Tidsskr Nor Laegeforen.

[20] Selin L, Almstrom E, Wallin G, Berg M (2009). Use and abuse of oxytocin for augmentation of labor. Acta Obstet Gynecol Scand.

[21] Brunstad Anne TE (2010). Jordmorboka.

[22] WHO United Nations Population Fund, UNICEF. Integrated Management of Pregnancy and Childbirth. Pregnancy, Childbirth, Postpartum and Newborn Care: A guide for essential practice, 3rd edition. 26561684

[23] Friedman E (1954). The graphic analysis of labor. Am J Obstet Gynecol.

[24] Zhang J, Landy HJ, Branch DW, Burkman R, Haberman S, Gregory KD, Hatjis CG, Ramirez MM, Bailit JL, Gonzalez-Quintero VH (2010). Contemporary patterns of spontaneous labor with normal neonatal outcomes. Obstet Gynecol.

[25] Zhang J, Troendle J, Mikolajczyk R, Sundaram R, Beaver J, Fraser W (2010). The natural history of the normal first stage of labor. Obstet Gynecol.

[26] Friedman EA (1978). Classic pages in obstetrics and gynecology. The graphic analysis of labor. Emanuel A. Friedman. Am J Obstet Gynecol.

[27] Philpott RH, Castle WM (1972). Cervicographs in the management of labour in primigravidae. II. The action line and treatment of abnormal labour. J Obstet Gynaecol Br Commonw.

[28] Zhang J, Troendle JF, Yancey MK (2002). Reassessing the labor curve in nulliparous women. Am J Obstet Gynecol.

[29] Vahratian A, Troendle JF, Siega-Riz AM, Zhang J (2006). Methodological challenges in studying labour progression in contemporary practice. Paediatr Perinat Epidemiol.

[30] Norsk gynekologisk forening. Veileder i fødselshjelp; 2014, Chapter 34. http://legeforeningen.no/Fagmed/Norsk-gynekologisk-forening/Veiledere/veileder-i-fodselshjelp-2014.

[31] Robson MS (2001). Can we reduce the caesarean section rate?. Best Pract Res Clin Obstet Gynaecol.

[32] Campbell MK, Piaggio G, Elbourne DR, Altman DG, Group C (2012). Consort 2010 statement: extension to cluster randomised trials. BMJ (Clinical research ed).

[33] Dencker A, Taft C, Bergqvist L, Lilja H, Berg M (2010). Childbirth experience questionnaire (CEQ): development and evaluation of a multidimensional instrument. BMC Pregnancy Childbirth.

[34] Section for Applied Clinical Research-NTNU. http://www.ntnu.edu/dmf/akf/skanning. Accessed 15 Nov 2014.

[35] USIT-Center for Information Technology Services, University of Oslo, Norway. http://www.uio.no/tjenester/it/applikasjoner/nettskjema/mer-om/. Accessed Nov 2014.

[36] Hayes RJ, Bennett S (1999). Simple sample size calculation for cluster-randomized trials. Int J Epidemiol.

[37] Bennett S, Parpia T, Hayes R, Cousens S (2002). Methods for the analysis of incidence rates in cluster randomized trials. Int J Epidemiol.

[38] Neal JL, Lowe NK (2012). Physiologic partograph to improve birth safety and outcomes among low-risk, nulliparous women with spontaneous labor onset. Med Hypotheses.

[39] Zhang J, Troendle J, Reddy UM, Laughon SK, Branch DW, Burkman R, Landy HJ, Hibbard JU, Haberman S, Ramirez MM (2010). Contemporary cesarean delivery practice in the United States. Am J Obstet Gynecol.

